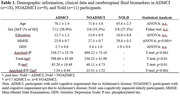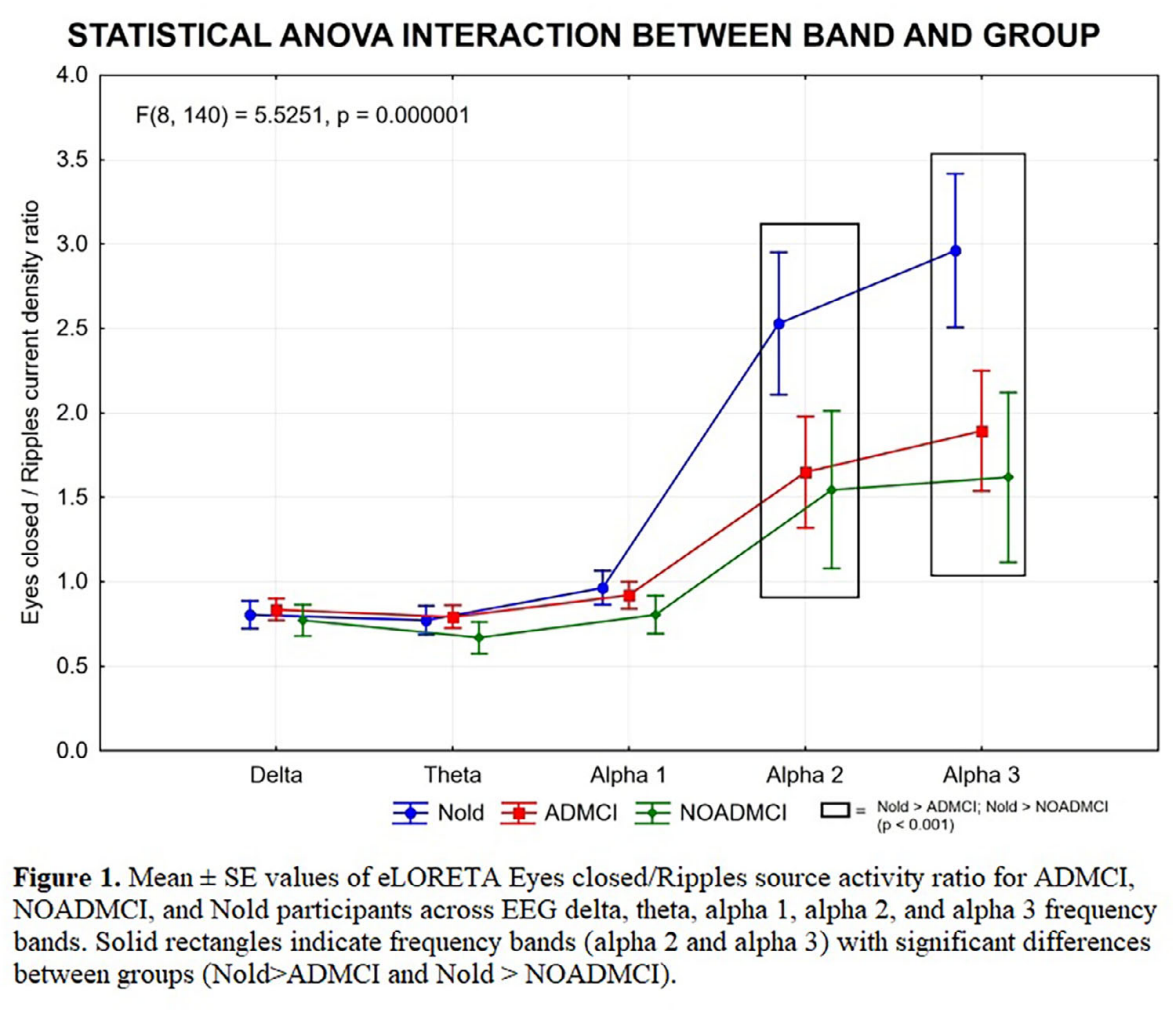# EEG Alpha Rhythm Abnormalities During the Transition from Wakefulness to Light Sleep in Patients with Mild Cognitive Impairment Due to Neurodegenerative Diseases

**DOI:** 10.1002/alz.092543

**Published:** 2025-01-09

**Authors:** Matteo Carpi, Enrico Michele Salamone, Dharmendra Jakhar, Roberta Lizio, Giuseppe Noce, Susanna Lopez, Bahar Güntekin, Görsev Yener, John‐Paul Taylor, Dario Arnaldi, Claudio Del Percio, Claudio Babiloni

**Affiliations:** ^1^ Sapienza University of Rome, Rome Italy; ^2^ IRCCS Synlab SDN, Naples Italy; ^3^ Istanbul Medipol University, Istanbul Turkey; ^4^ Izmir University of Economics, Faculty of Medicine, Balçova, Izmir Turkey; ^5^ Newcastle University, Newcastle upon Tyne UK; ^6^ IRCCS Ospedale Policlinico San Martino, Genoa Italy; ^7^ University of Genoa, Genoa Italy; ^8^ Sapienza University of Rome, Rome, Rome Italy; ^9^ San Raffaele Cassino, Cassino Italy

## Abstract

**Background:**

Vigilance and sleep disturbances in Alzheimer’s and related diseases, even at the stage of mild cognitive impairment (MCI), have been extensively documented, showing abnormal daytime naps and alterations in the sleep‐wake cycle. However, the EEG correlates of the transition from wakefulness to light sleep have not yet been compared between MCI patients due to Alzheimer’s vs. other neurodegenerative diseases. Therefore, it is unclear whether there are specific features of these correlates in Alzheimer’s disease (AD) patients. This issue was investigated in the present study for the first time.

**Method:**

Data were analyzed from an international database (https://www.pdwaves.eu/) comprising neuropsychological tests, long resting‐state EEG recordings, cerebrospinal fluid biomarkers, and MRI. For this study, 18 ADMCI patients, 9 NOADMCI patients, and 11 age‐ and sex‐matched healthy (Nold) participants showing transitions from wakefulness to light sleep during the EEG recording were selected based on the evaluation of two researchers blinded to the diagnoses. EEG periods were classified into four classes according to Hori et al.’s criteria: eyes open, eyes closed, EEG flattening, and EEG ripples (the last two representing drowsiness/light sleep stages). Individual alpha frequency peak (IAF) was obtained and used to determine EEG delta, theta, and alpha (1, 2, and 3) bands. Regional EEG cortical sources (Frontal, Parietal, and Occipital) were estimated using eLORETA freeware and the ratio of the source current density for the eyes closed and ripples conditions (EC/R) was considered as the EEG correlate of sleep‐wake transitions.

**Result:**

Participants’ characteristics are reported in Table 1. The ANOVA showed a 2‐way interaction between Group and Band (p<.001), unveiling reduced EC/R source activity in the alpha 2 (p‐values<.001) and alpha 3 (p‐values<.001) bands in the ADMCI and NOADMCI groups (Figure 1). No differences in EEG source activity were found between the ADMCI and NOADMCI groups (p>.05).

**Conclusion:**

The preliminary results of the present study showed widespread abnormalities in the cortical neurophysiological mechanisms oscillating at alpha frequencies in patients with Alzheimer’s and related diseases at the stage of MCI, but no differences between Alzheimer’s and non‐Alzheimer’s diseases. Future studies should cross‐validate these findings with larger clinical populations and test their relationship with night sleep disorders.